# Two Matrix Metalloproteinase Inhibitors from *Scrophularia Striata* Boiss

**Published:** 2014

**Authors:** Hamid Reza Monsef–Esfahani, Ahmad Reza Shahverdi, Mohammad Reza Khorramizadeh, Mohsen Amini, Reza Hajiaghaee

**Affiliations:** a *Department of Pharmacognosy, Faculty of Pharmacy, Tehran University of Medical Sciences, Tehran, Iran.*; b *Department of Pharmaceutical Biotechnology and Pharmaceutical Research Center, Faculty of Pharmacy, Tehran University of Medical Sciences, Tehran, Iran*^*.*^; c *Department of Pathobiology, School of Public Health, Tehran University of Medical Sciences, Tehran, Iran. *; d *Department of Medicinal Chemistry, Faculty of Pharmacy, Tehran University of Medical Sciences, Tehran, Iran. *; e *Drug Design and Development Research Center, Tehran University of Medical Sciences, Tehran, Iran.*; f *Pharmacognosy and Pharmaceutics Department of Medicinal plants Research Center, Institute of Medicinal Plants, ACECR, Karaj, Iran.*

**Keywords:** *Scrophularia striata*, Nepitrin, Acteoside, Wehi-164, Zymoanalysis, MMPs

## Abstract

Many species belonging to the *Scrophularia* genus have been used since ancient times as folk remedies for many medical conditions such as scrofulas, scabies, tumors, eczema, psoriasis, inflammations.

The aim of this study was to characterize the matrix metalloproteinases (MMPs) inhibitor compounds of the *Scrophularia striata* extract by bio-guide fractionation.

The aerial parts of *S. striata* were collected and different extracts were sequentially prepared with increasingly polar solvents. The MMPs inhibitory activity of the crude extract and its fractions were evaluated by the Zymoanalysis method. The pure compounds were purified from the active fraction by chromatography methods. Chemical structures were deduced by nuclear magnetic resonance and mass spectrometry.

Two active compounds (acteoside and nepitrin) were identified by bio-guide fractionation. The inhibitory effects of nepitrin and acteoside at 20 µg/mL were about 56 and 18 percent, respectivly. The inhibitory effects of acteoside at 80 µg/mL were increased to about 73 percent. In summary, the results suggest that nepitrin effectively inhibited MMPs inhibitory activity at low concentrations, whereas acteoside showed inhibition at high concentrations.

## Introduction

Metastasis, the major cause of cancer mortality, is a complex phenomenon in which tumor cells invade surrounding tissues, penetrate blood vessels and exit vessels at distant sites to form secondary tumors (1). Most investigators unanimously agree that matrix metalloproteinases (MMPs) are critical enzymes involved in many aspects of cancer, including tumor growth, invasion, metastasis (2-3-4) and neovascularization (5-6).

MMPs are a family of zinc-dependent endoproteinases that play pivotal roles in the dynamic remodeling of the extracelluar matrix. Based on substrate preference and structural homology, MMPs are sub-classified by their functional groups into collagenases, gelatinases, stromelysins, matrilysins, membrane type MMPs (MT-MMPs) and other non-classified MMPs (7-8).

The genus *Scrophularia*, consisting of about 300 species, is one of the most important genera belonging to the Scrophulariaceae. Many species belonging to this genus have been used since ancient times as folk remedies for many medical conditions (scrofulas, scabies, tumors, eczema, psoriasis, inflammations, *etc.*) (9-10). For example, dried roots of *S. ningpoensis* Hemsl are used as antipyretic, febrifuge and antibacterial, as a remedy for evening fever, erythema, mouth dryness, constipation, prurigo, furunculosis, sore throat, ulcerous stomatitis, tonsillitis and in the treatment of cancer (11-12). *S. deserti* is used in traditional medicine as an antipyretic, a remedy for kidney diseases and tumors and lung cancer (13).

Hajiaghaee *et al.* (2007) reported that a total extract of *S. striata* at 80 µg/mL concentration moderately inhibited growth of the wehi-164 cell line (37%), whereas at lower doses (down to 10 µg/mL) its cytotoxicity was negligible and the cell viability percentage was more than 70% (14).

In the present paper, the inhibitory effect of *S. striata* was studied using bio-guide fractionation. The active substances and their chemical structures were deduced by nuclear magnetic resonance (NMR) and mass spectrometry.

## Experimental

*Chemical and reagents*

Silica gel (Merck), Sephadex LH-20 (Fluka) and polyamide 6 (Fluka) were used as stationary phases in column chromatography fractionations. The fibrosarcoma cell line (wehi-164) was obtained from the National Cell Bank of Iran (NCBI), Pasteur Institute of Iran, Tehran, Iran. Daru-Pakhsh Co. supplied piroxicam. 

*General*

The ^1^H and ^13^C NMR spectra of the isolated compound were measured in CD_3_OD at 500 and 125 MHz, respectively, using a Bruker AC 500 Spectrophotometer (Germany). Mass spectra were taken on a Finnigan TSQ- Mat 70 (70 eV) Spectrometer.

*Plant material*

The aerial parts of *S. striata* were collected from plants growing in the northeastern part of Iran, in the Ruin region (1350 m above sea level) in May 2006 and were dried at room temperature. A sample was authenticated by Dr F. Attar, and a voucher specimen was preserved in the Faculty of Sciencesۥ Herbarium at Tehran University, Tehran, Iran (TUH no. 36501).

*Extraction and isolation*

Different extracts were sequentially prepared with increasingly polar solvents using 1 Kg of the dried and powdered aerial parts of the plant and 5 L of each solvent. The resulting extracts consisted of: Petroleum ether (6.6 g dry weight corresponding to 0.6%), chloroform (11.4 g dry weight corresponding to 1.1%), ethyl acetate (12.8 g dry weight corresponding to 1.2%) and 80% methanol extract (16.4 g dry weight corresponding to 1.6%).

The 80% methanol extract was subjected to polyamide column chromatography and eluted by water followed by water-methanol mixes of decreasing polarity. Six main fractions were obtained (a- f). TLC analysis was performed on silica gel using ethyl acetate- methanol-water-acetic acid in various proportions as the mobile phase. Compounds were visualized under UV light (254 and 365 nm) or by spraying the plates with anisaldehyde-sulfuric acid reagent (15). Fractions b and d significantly inhibited MMPs activity. Re- chromatography of the fractions b and d on Sephadex LH-20 (methanol), yielded 32 mg nepitrin and 28 mg acteoside as active compounds.

*Cell culture*

The wehi-164 cells were seeded in 96-well tissue culture plates. Cells were maintained in an RPMI-1640 medium supplemented with 5% fetal calf serum, plus antibiotics, at 5% CO_2_ and saturated humidity.

*Dose- response analysis*

Two-fold dilution of plant extracts, column fractions and the reference drug (piroxicam) were added to triplicate samples of cultured cells. Untreated cells were used as controls. Cells were cultured overnight, and then subjected to a colorimetric assay. Cytotoxicity was expressed as the percentage of viable cells at different sample concentrations. IC_50_ was calculated as the dose at which 50% of the cells died relative to the untreated cells. The corresponding supernatants of the cultured cells were used for zymoanalysis.

*Colorimetric assay*

In the cytotoxicity assay, cells in the exponential phase of growth phase were incubated for 24 h at 37 ºC with 5% CO_2_ with a serial dilution of samples. Cell proliferation was evaluated by a modified crystal violet colorimetric assay (16). After each experiment, the cells were washed with ice-cold PBS and fixed in a 5% formaldehyde solution. Fixed cells were stained with 1% crystal violet and the stained cells were then layered and solubilized with a 33.3% acetic acid solution. The density of the purple product that developed was measured at 580 nm.

*Zymoanalysis*

This technique was used to detect gelatinase (collagenase type IV or matrix metalloproteinase type-2, MMP-2) and MMP-9 in conditioned media (17). Briefly, aliquots of conditioned media were subjected to electrophoresis in a gelatin-containing polyacrylamide gel, in the presence of sodium dodecyl sulfate (SDS) under non-reducing conditions. After electrophoresis, SDS was removed by repeated washing with Triton X-100. The gel slabs were incubated overnight at 37 ºC in a gelatinase-activating buffer and subsequently stained with Coomassie Brilliant Blue R 250 (Sigma, MA). After intensive destaining, proteolytic areas appeared as clear bands against a blue background. Using a gel documentation system, quantitative evaluations of both the surface and intensity of the lysis bands, on the basis of grey levels, were compared with the untreated control wells and expressed as a percentage of the "relative expression" of gelatinolytic activity. The IC_50_ for the MMPs inhibitory effect was calculated as the doses at which 50% MMPs inhibition occurred relative to untreated control cells.

*Statistical analysis*

The differences in cell cytotoxicity and gelatinase zymography were compared using the Studentۥs t-test. Values of p < 0.05 were considered significant.

## Results and Discussion

**Figure 1:**
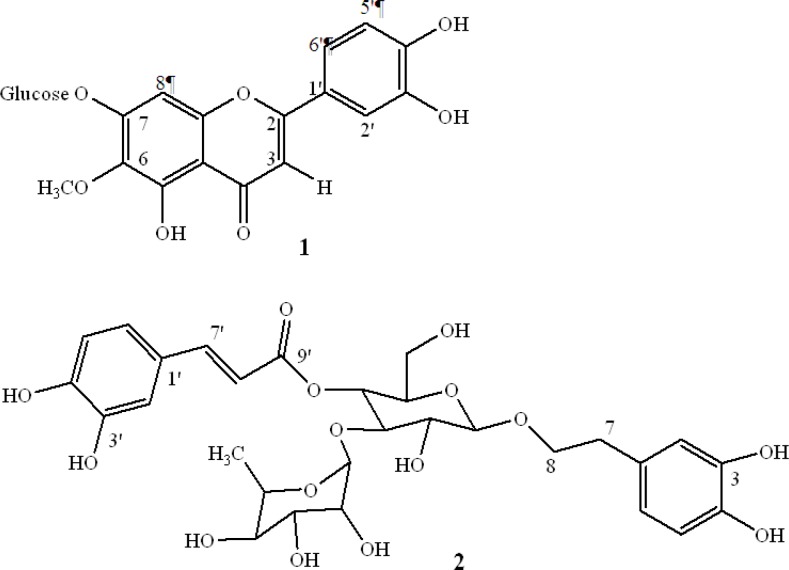
The structure of bioactive compounds isolated from *Scrophularia striata*: (1) nepitrin, (2) acteoside

In a previous study, we showed inhibitory effects of a total extract and sequential extracts of *S. striata*. An 80% methanol extract, compared with other extracts significantly inhibited MMPs activity and showed low cytotoxicity. The activity of different extracts and fractions are summarized in Table 1. The 80% methanol solution was subjected to activity-guided fractionation on a polyamide column and eluted with a water–methanol mixture. The active fractions were subjected to further purification on a Sephadex LH-20 column, with the result that two compounds with MMPs inhibitory activity were obtained (Figure 1).

**Table 1:** Cell viability and MMPs activity of different extracts and fractions of *Scrophularia striata* on Wehi-164.

**Cell viability (%)** ^a^	**Relative MMPs activity (%)** ^a^	**Concentration (µg/mL)**	**Sample**
99.98 ± 1.20	100.2 ± 0.43	-	Control
87.54 ± 2.21***	95.26 ± 0.40***	10	Reference drug
85.46 ± 1.82***	85.37 ± 0.35***	20	(piroxicam)
70.36 ± 2.30***	80.47 ± 0.37***	40	
50.27 ± 2.45***	65.36 ± 0.40***	80	
			
85.96 ± 1.09***	44.44 ± 0.53***	10	Total extract
76.51 ± 5.10***	24.55 ± 0.40***	20	
69.34 ± 2.10***	23.30 ± 0.35***	40	
63.85 ± 0.74***	17.24 ± 0.36***	80	
			
88.78 ± 0.79***	52.82 ± 0.31***	10	Petroleum ether
81.91 ± 1.91***	49.12 ± 0.28***	20	extract
75.09 ± 1.81***	33.20 ± 0.28***	40	
70.36 ± 0.65***	32.75 ± 0.18***	80	
			
72.06 ± 1.62***	87.37 ± 0.29***	10	Chloroform
60.18 ± 3.45***	78.31 ± 0.25***	20	extract
30.80 ± 2.04***	51.26 ± 0.31***	40	
24.62 ± 0.82***	19.59 ± 0.31***	80	
			
97.97 ± 0.42	94.53 ± 0.29***	10	Ethyl acetate
74.57 ± 5.57***	88.29 ± 0.31***	20	extract
27.41 ± 0.78***	65.89 ± 0.31***	40	
25.06 ± 0.84***	36.03 ± 0.39***	80	
			
96.11 ± 0.76	55.65 ± 0.22***	10	80% methanol
91.33 ± 0.97**	40.76 ± 0.26***	20	extract
74.12 ± 1.18***	35.29 ± 0.21***	40	
60.16 ± 4.53***	17.25 ± 0.24***	80	
			
97.98 ± 0.19	75.85 ± 0.44***	10	Fraction b
82.72 ± 0.56***	63.59 ± 1.47***	20	
76.48 ± 1.15***	57.16 ± 1.42***	40	
59.42 ± 1.17***	52.64 ± 0.86***	80	
			
75.86 ± 0.56***	84.94 ± 0.31***	10	Fraction d
69.76 ± 1.13***	81.34 ± 0.63***	20	
68.44 ± 1.12***	73.28 ± 1.24***	40	
64.18 ± 0.40***	45.92 ± 1.70***	80	

* p < 0.05.

** p < 0.01.

*** p < 0.001.

^a^ Each data point was compared with control group and statistical analysis was performed with the Student's t-test.

The chemical structure confirmation of the components from the *S. striata* methanol extract was accomplished by comparing the obtained ^1^H and ^13^C NMR data to those previously published.

Compound 1 was obtained as yellow needles; EI-MS, m/z 315 [M - (Glu)]. ^1^H NMR (CD_3_ OD, 500 MHz) δ: 6.6 (^1^H, s, H_3_), 6.5 (^1^H, s, H_8_), 7.5 (^1^H, d, *J* = 2.1 Hz, H_2'_), 6.9 (^1^H, d, *J* = 8.5 Hz, H_5'_), 7.4 (^1^H, dd,* J* = 8.5, 2.1 Hz, H_6'_), 3.9 (3H, s, OCH_3_), 5.1 (^1^H, d,* J *= 7.3 Hz, H_1"_), 3.01-3.7 (5H, m, H_2"_, H_3"_, H_4"_ , H_5"_, H_6"_). ^13^C NMR (CD_3_OD) δ: 56.7 (OCH_3_), 62.2 (C_6"_), 70.9 (C_4"_), 75.4 (C_2"_), 77.8 (C_3"_), 78.7(C_5"_), 96.0 (C_8_), 103.7 (C_1''_), 105 (C_3_), 106.7 (C_10_), 110.8 (C_2'_), 116.8 (C_5'_), 121.7 (C_6'_), 123.7 (C_1'_), 130.6 (C_6_), 149.5 (C_3'_), 151.3 (C_4'_), 152.1 (C_5_), 153.8 (C_9_), 155.5 (C_7_), 166.2 (C_2_), 184.0 (C_4_). These spectral data were in agreement with the reported literature. Therefore, compound 1 was identified as nepitrin (18).

Compound 2 was isolated as an amorphous powder; ^1^H NMR (CD_3_OD) δ: 1.07 (3H, d, *J* = 6.0 Hz, H_6"'_), 2.7 (2H, m, H_7_), 4.3 (^1^H, d, *J* = 8.0 Hz, H_1"_), 4.9 (^1^H, t, *J* = 10.0 Hz, H_4"_), 5.1 (^1^H, s, H_1"'_), 6.2 (^1^H, d, *J *= 16.5 Hz, H_8'_), 6.5 (^1^H, dd, *J* = 7.5, 1.5 Hz, H_6_), 6.6 (^1^H, d, *J* = 7.5 Hz, H_5_), 6.68 ( ^1^H, d, *J* = 1.5 Hz, H_2_), 6.7 (^1^H, d, *J* = 8.5 Hz, H_5'_), 6.9 (1H, d, *J* = 8.5 Hz, H_6'_), 7.0 (^1^H, s, H_2'_), 7.5 (^1^H, d, *J* = 16.5 Hz, H_7'_). ^13^C NMR ( CD_3_OD) δ: 18.1(C_6"'_), 36.5 (C_7_), 62.4 (C_6"_), 70.4(C_5"'_), 70.6 (C_4"_), 72.0 (C_3"'_), 72.2 (C_8_), 72.3 (C_2"'_), 73.8 (C_4"'_), 76.0 (C_5"'_), 76.2 (C_2"_), 81.6(C_3"_), 102.9 (C_1"'_), 104.2 (C_1"_), 114.5 (C_8'_), 115.2 (C_2'_), 116.41 (C_5_), 116.8 (C_5'_), 117.2 (C_2_), 121.3 (C_6_), 123.6 (C_6'_), 127.5 (C_1'_), 131.6 (C_1_), 144.6 (C_4_), 146.1 (C_3_), 147.0 (C_3'_), 148.1 (C_7'_), 150.2 (C_4'_), 168.4 (C_9'_) agree well with previously reported data (19). Therefore, compound 2 was identified as acteoside.

**Figure 2:**
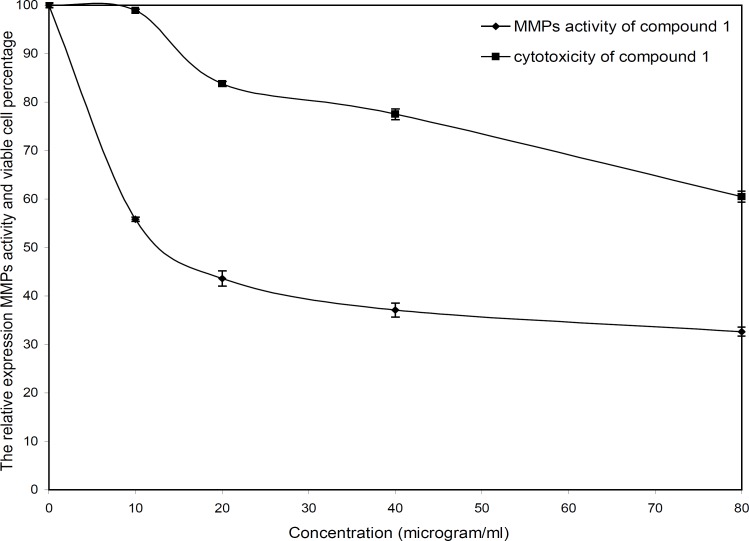
Cytotoxicity and MMPs analysis of compound 1 (nepitrin) from *Scrophularia* *striata*

**Figure 3:**
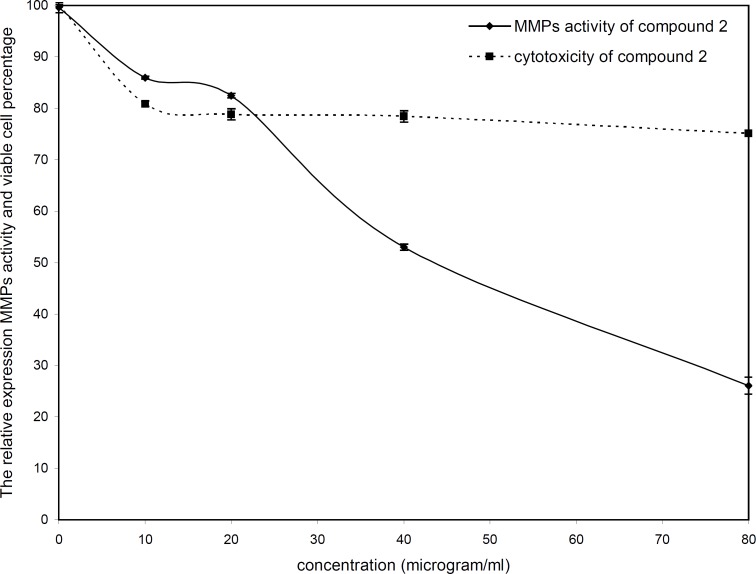
Cytotoxicity and MMPs analysis of compound 2 (acteoside) from *Scrophularia* *striata*

The cytotoxicity and inhibitory effect of the active compounds of *S. striata* were evaluated *in-vitro* at four doses (10, 20, 40 and 80 µg/mL) against the wehi-164 cell line. Both active compounds showed a direct dose-response as higher concentrations led to higher toxicity and inhibitory effects (Figure 2 and 3).

The invasion of wehi-164 cells was significantly inhibited at lower concentrations of nepitrin. The inhibitory effects of nepitrin at doses of 10 and 20 µg/mL were about 44 and 56 percent, respectively. Nepitrin at 20-80 µg/mL concentrations moderately inhibited MMPs activity, whereas at lower doses (down to 20 µg/mL) its anti-invasive activity was substantial. Its cytotoxicity at lower doses was negligible and there was no significant difference between the control (0 µg/mL) and 10 µg/mL. The IC_50_ values for cytotoxicity and zymoanalysis observed for the cell line are reported in Table 1.

**Table 2:** IC_50_ of values for cytotoxicity and MMPs inhibition by total extract, reference drug, fractions and isolated compounds from *Scrophularia striata*

** IC**_50_ ** (µg/mL)**		**sample**
Cytotoxicity	Zymoanalysis	
97.59	13.68	Total extract
75.98	98.72	Reference drug (piroxicam)
86.62	71.28	Fraction b
107.11	72.74	Fraction d
87.94	32.90	Compound 1
116.51	51.73	Compound 2

The inhibitory effect of acteoside at lower doses (down to 20 µg/mL) was less than 18%, whereas at 40 and 80 µg/mL inhibition increased to about 47 and 73 percent, respectively. The viability percentage of acteoside at 10 µg/mL was about 81% and its cytotoxicity at 20, 40 and 80 µg/mL was negligible. The IC_50_ value for zymoanalysis was calculated as 51.73 µg /mL.

In the present study, isolation of two MMP inhibitors was obtained using bioactivity-guided fractionation of aerial parts of *S. striata*. Nepitrin effectively inhibited MMPs activity at low concentrations, whereas acteoside showed inhibition at high concentrations. Future work to extend these results should investigate the effect of these compounds on different cell lines, to gain a better perspective of their properties.
